# Applications of Machine Learning Predictive Models in the Chronic Disease Diagnosis

**DOI:** 10.3390/jpm10020021

**Published:** 2020-03-31

**Authors:** Gopi Battineni, Getu Gamo Sagaro, Nalini Chinatalapudi, Francesco Amenta

**Affiliations:** 1 Center for Telemedicine and Tele pharmacy, School of Medicinal and Health Sciences Products, University of Camerino, Via Madonna Della carceri 9, 62032 Camerino, Italy; getugamo.sagaro@unicam.it (G.G.S.); nalini.chintalapudi@unicam.it (N.C.); francesco.amenta@unicam.it (F.A.); 2 Research Department, International Medical Radio Center Foundation (C.I.R.M.), 00144 Roma, Italy

**Keywords:** chronic diseases, prediction models, pathologies, accuracy, disease classification

## Abstract

This paper reviews applications of machine learning (ML) predictive models in the diagnosis of chronic diseases. Chronic diseases (CDs) are responsible for a major portion of global health costs. Patients who suffer from these diseases need lifelong treatment. Nowadays, predictive models are frequently applied in the diagnosis and forecasting of these diseases. In this study, we reviewed the state-of-the-art approaches that encompass ML models in the primary diagnosis of CD. This analysis covers 453 papers published between 2015 and 2019, and our document search was conducted from PubMed (Medline), and Cumulative Index to Nursing and Allied Health Literature (CINAHL) libraries. Ultimately, 22 studies were selected to present all modeling methods in a precise way that explains CD diagnosis and usage models of individual pathologies with associated strengths and limitations. Our outcomes suggest that there are no standard methods to determine the best approach in real-time clinical practice since each method has its advantages and disadvantages. Among the methods considered, support vector machines (SVM), logistic regression (LR), clustering were the most commonly used. These models are highly applicable in classification, and diagnosis of CD and are expected to become more important in medical practice in the near future.

## 1. Introduction 

Artificial intelligence (AI) is defined as the technology that uses computer knowledge to represent intelligent behavior with nominal human involvement, and machine learning (ML) is considered as a subset of AI techniques. Usually, this kind of intelligence is commonly acknowledged as having begun with the innovation of robotics [1]. With the rapid growth of electronic speeds and programming, computers may display intelligent behavior similar to that of humans in the near future. This is because of the large advancements happening in contemporary ideas in the development of AI [2]. Artificial intelligence can be defined as human intelligence which is performed by machines. In computer science, it is defined as the machine’s capacity to emulate intelligent behavior by itself, using nothing but ML [3]. 

The applications of AI in medicine are developing quickly. In 2016, AI projects coupled with medicine drew in more speculation from the global economy than other projects [4]. In medicine, AI refers to the utilization of automated diagnosis processes and the treatment of patients who require care. Increased AI utilization in prescription will allow a considerable amount of the role to be automated, opening up medicinal experts’ time to be used in performing different obligations, ones that cannot be automated. As such, this technology promises progressively significant utilization in the field of human resources (HR). 

In general, ML is categorized as supervised (i.e., consists of output variables that are predicted from input variables) [5] or unsupervised (i.e., deals with clustering of different groups for a particular intervention). ML is used to determine complex models, and extract medical knowledge, exposing novel ideas to practitioners, and specialists [2]. In clinical practice, ML predictive models can highlight enhanced rules in the decision-making regarding individual patient care. These are also capable of autonomous diagnosis of different diseases under clinical regulations [4,6,7,8]. In [9], the incorporation of these models in drug prescription can save doctors and offer new medical opportunities in pathology identification. 

With ML models, it can also be possible to improve quality of medical data, reduce fluctuations in patient rates, and save in medical costs. Therefore, these models are frequently used to investigate diagnostic analysis when compared with other conventional methods [10]. To reduce the death rates caused by chronic diseases (CDs), early detection and effective treatments are the only solutions [11]. Therefore, most medical scientists are attracted to the new technologies of predictive models in disease forecasting [12]. These new advancements in medical care have been expanding the accessibility of electronic data and opening new doors for decision support and productivity improvements [13]. ML methods have been effectively utilized in the computerized interpretation of pneumonic capacity tests for the differential analysis of CDs. It is expected that the models with the highest accuracies could gain large importance in medical diagnosis. 

Due to the low-progress nature of CDs, it is important to make an early prediction and provide effective medication. Therefore, it is essential to propose a decision model which can help to diagnose chronic diseases and predict future patient outcomes. While there are many ways to approach this in the field of AI, the present study focuses distinctly on ML predictive models used in the diagnosis of CDs, which highlights the importance of this study. In this study, we conducted a systematic literature review of different state-of-art of predictive models, and our significant contribution in this paper is to develop comparative model analysis to propose model optimization. In comparison to the conventional data analysis techniques, this review article will able to find promising results that enhance the quality of patient data and analysis of specific items that are related to ML algorithms in medical care.

## 2. Methods

### 2.1. Search Strategy 

The systematic literature search was conducted through the libraries of PubMed (Medline) and Cumulative Index to Nursing and Allied Health Literature (CINAHL). Keywords like ‘chronic diseases’, ‘predictive models’, ‘ML in CD diagnosis’, and ‘model classifiers’ were used during the document search. The search was conducted in January 2020 and resulted in 453 documents. The documents were filtered based on its publication dates ranging from 2015 to 2019 to evaluate the latest literature on ML classifiers in CD prediction. 

### 2.2. Selection Criteria 

The title and abstract of the individual articles were retrieved based on the mentioned search terms. Finally, a few of the items were found to be eligible to fulfill the research objectives. This research only describes predictive models used to perform CD diagnosis and does not concentrate on overall trends in AI medicine. Further article revision was conducted to filter the duplicates between the two databases. Moreover, the inclusion and exclusion criteria of our review were based on time, methodological quality and language. Reports and other studies published before 2015 were excluded as outside the limitations on the timeframe of this study. The inclusion criteria used in Pub Med and CINAHL are as follows: free full text, English, original papers and research outcomes. We excluded 276 items among the total search documents because of duplication. The remaining 177 were screened to match the methodologies related to the current research topic. 

### 2.3. Data Extraction

Data evaluation was conducted in two phases. In the first phase, depending on the inclusion criteria, 55 documents were identified for extended revisions. In the second phase, two individual researchers (GB and GGS) were equally distributed for quality check. As discussed, the proposal of a precise model in CD diagnosis was considered as the main focus of this paper. Therefore, articles were extracted based on the authors’ information, the study design of sampling pattern and method types, and diagnostic criteria. The analysis of each article was individually revised and recorded.

### 2.4. Quality Evaluation

**Figure 1:**
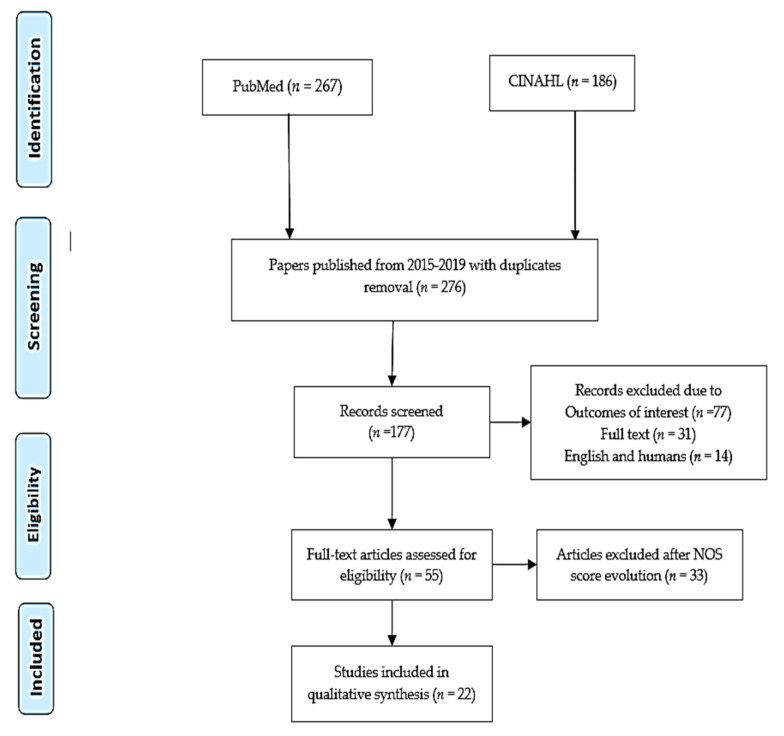
Preferred reporting items for systematic reviews and meta-analyses (PRISMA) diagram [15].

Quality assessment check was accomplished by the adoption of the Newcastle–Ottawa Scale (NOS), which is a renowned method in the assessment of study relevance and research interest [14]. The quality of each published article was evaluated as weak (0–4), moderate (5–6), or strong (7–9). Each selected study score was recorded in separate excel sheets to compute whether an individual paper was suitable or not for this review. Ultimately, 22 studies were selected, which are in line with the predictive models in the CD diagnosis (Figure 1). Based on their content, the selected papers were tabled into predictive models used in CD identification (Table 1) and pathologies with model usage, along with their strengths and limitations (Table 2).

## 3. Results

### 3.1. Predictive Models Applied in Diagnosis of CD 

From Table 1, it is evident that about 45% of studies used SVM models, 23% of the studies used K-Nearest Neighbor (KNN), and Naïve Bayes (NB) models, 18% of studies applied LR, and 14% of studies applied random forest (RF) models in the CD diagnosis. Regression-based ML models were largely used to predict liver, gas chromatography, and pathological changes. Two studies successfully implemented the random forest (RF) model to do a prediction of the liver fibrosis stages [16,19]. These studies also applied the linear regression (LR) statistical analysis to understand the relationship of image parameter and liver fibrosis stages. The results highlight that RF models are better at identifying the liver fibrosis index (LFI) degree than other statistical approaches [16]. A review of 427 patients on hepatitis-C produced better predictions through the decision trees [17], and multilayer perceptron (MLP) neural networks were best in predicting late-stage liver fibrosis.

In [20], COPD patient’s data were analyzed by Bayesian network models. The usage of support vector machines (SVM), LR, Bayesian network, and K-Nearest Neighbor (KNN) models is useful in forecasting the aggravating events of COPD patients [20,21,22]. Among them, SVM models show better accuracy in predicting exacerbations and COPD detection. In addition, artificial neural networks (ANN) and LR models can effectively be used to understand whether a patient is diabetic or not [23]. In [24], scholars estimated the glomerular filtration rate of the kidney can be done through ensemble models. 

### 3.2. Model Accuracies along with advantages and limitations 

Model accuracy is defined as a percentage of true predictions from total predictions. From Table 2, it is evident that diabetic predictions show an accuracy of 73.1–91.6% [23]. Cardiac diseases produce a prediction accuracy of 84–91% [34], while in the prediction of liver diseases, NB, RF, KNN, SVM, and NN models produce an accuracy in a range of 78.1–82.7%. In particular, RF and NN models are found to have the highest accuracy. In nervous system pathologies, the LR model with feature extraction techniques has identified reasons for depression with accuracy between 72% and 80% [48]. KNN models could be better used to identify disease patterns [49]. In Fibromyalgia, these have the capability to classify pain, clinical usage, and symptom severity [36]. In pulmonary diseases, Bayesian models produced low accuracy range in between 62.3% and 76.1%, because these are not recommended in high dimensional data sets. In contrast, ANN models produced the highest accuracy of 76% in kidney diseases, since they can detect the possible connection between classification variables [50].

Despite the above results, it is still debatable matter on the existence of particular microbial profiles for distinct periodontal conditions. Studies conducted on the ML model development in classifying patient data based on bacterial species [35] have shown that SVM with kernel methods are more helpful. At the same time, dementia is one of the chronic diseases that happen in older people, and, in particular, Alzheimer’s is associated with 60–70% of dementia cases. AD prediction through ML models concluded that prediction accuracy depends on the data type and model input [28,31]. These studies with the Disease State Index (DSI) technique produced an accuracy of 79%. All the mentioned studies found that age, cognition, subjective memory complaints, and vascular factors were input features, which can affect the chances of dementia. Therefore, it is understandable that dataset type, input features, and user outcomes can differ by individual study and no model can predict diseases with 100% accuracy.

## 4. Discussion

The present study analyses the distinct prediction models of machine learning in the diagnosis of chronic diseases. Sometimes, it could be hard to propose the best learning method in disease predictions since it depends on dataset size and user access. Supervised machine learning (SML) approaches are followed in the highest number of studies, with the integration of easy and simple predictive modeling. The implementation of these models in clinical practice certainly can help to provide better health services and enhance specialist decision-making. 

It is rudimentary to confirm the different algorithms based on a specific problem, and review studies could help to analyze the performance and determine optimal machine learning models. Before machine learning, recommendations for practice in medicine development depended on individual studies. Therefore, it is affecting the data science because all this medical information is coming from different platforms and people. Due to contemporary trends in computational models, healthcare services are quickly transformed by having the ability to record large amounts of patient data. However, it is highly impossible to analyze huge medical records with human knowledge. On the other hand, with the evolvement of big data in biomedical and medicinal service networks, accurate analysis of medical data becomes possible that could improve patient care [51]; if there is an unavailability of quality medical data, it could result in poor decision-making. Eventually, machine-learning techniques can able to find clear data patterns that can empower health experts in clinical care such as precision medicine. As mentioned in Table 1, different studies have used different predictive models coupled with their results. However, a proper interpretation of medical data will not only help to recommend suitable machine learning models, but also for physicians in the provision of immediate medication. 

Aging is one of the significant difficulties facing in the western world, meaning that lowering the weight of continuous infection and enhancing life span is necessary [52,53]. Few researchers have anticipated the safety of each aging compound with a collection of deep neural network classifiers [53]. In addition, predictive models can help in active decision support, in particular if a couple of identical cases are accessible, or where diagnostic symptom knowledge is not precisely available [54]. In this study, we compared and discussed the recent advancements in ML methods used for disease predictions. Studies like pulmonary patient data classification demand the algorithm to anticipate discrete values by distinguishing the patient information either as an individual, or group [27]. The final clinical diagnosis depended on the integration of a full pulmonary function identification decided by medical expert choice. In the end, clinical diagnosis was classified into ten individual groups, which were validated by experts in the panel. Medical imaging data such as MRI, CT scans, and RTE images follow the classification type SVM models. In contrast, regression problems look at continuous data, and most of the adopted studies followed these model examples of pathologies in [16,30,33,48] gene expression [19], and others. 

On the other hand, unsupervised ML deals with a deep learning model containing a medical data set that it can handle without having a clear direction regarding what and how to proceed. The neural network attempts automatic detection of structured data and performs key feature extraction. Depending on the pathology type, it can follow different patterns like clusters that involve a group of particular information [37]. However, some models in machine learning can make immediate decisions on chronic diseases thanks to recent developments in AI. Our findings suggest that stimulating the power of these predictive models in the CDs diagnosis and in structuring medical data will empower medical experts or physicians that will result in a significant tendency decision making at medical centers. 

It is also evident that SVM and LR models significantly implemented in the large number of studies to do CD diagnosis. Sixteen studies were adopted these models especially for hepatitis B, COPD, diabetes, and others. An SVM model is popular among others to identify COPD from the beginning, and it could be greatly assisted in the relationship between doctor and patient. Bayesian networks and NB models help to forecast the diagnosis of asthma problems. These models encompass old patient records to look up clinical symptoms and footing on Bayesian networks to present the relationship individual case and diagnose future possible symptoms. The KNN algorithm is associated with five studies for diagnosis, forecasting, and to critically follow the CD’s stages with the help of different primary and secondary data.

The main limitation of the present study is most of the adopted literature on disease prediction or classification was adopted with supervised models, and it is important to adopt unsupervised (clustering) and deep learning (neural networks) models as well in future works.

## 5. Conclusions

The present study evaluated the studies associated with the diagnosis of chronic diseases. However, the implementation of correct methods or selection of the right models is a prerequisite to perform ideal decisions, as modern researchers are claiming that some ML models are compromised by enlarging contained datasets with malicious data that can have severe consequences. On the other hand, diagnosis limitations may lead to life-threatening attacks, and sometimes it might be a driving factor of fatality. In contrast, the wrong diagnosis prompts the skepticism in machine learning use, that can lead policy makers to avoid predictive model usage. Therefore, reviews on predictive models can provide evidence to propose excellent methods for the CDs diagnosis. 

In the future, AI techniques like ML, cognitive computing and deep learning may play a critical role in the interpretation of chronic diseases. However, researchers are progressively attracted by predictive model techniques in the advancement of health care. As new advancements in medical care are being established and are expanding the access to electronic data, this opens new doors to decision support and productivity improvements. These models are designed to emphasize the responsibility of patient care quality and cut down medical costs. 

## Figures and Tables

**Table 1:** Machine learning (ML) algorithms on different pathologies along with input features and outcomes measures.

CD Diagnosis	Study Type	Input Features	Outcomes	Models	Reference
Hepatic fibrosis	Cross-sectional	Age, sex and RTE images	Accuracy, Sensitivity, and Specificity	NB, RF, KNN, SVM, and NN	[16,17,18]
Chronic hepatitis B stages	Case study	Gene expressions	Precision and AU-ROC	RF, KNN, SVM	[19]
COPD exacerbation events	Retrospective	COPD symptoms	TP, FP, ROC	BN	[20]
Aggravating event identification of COPD	Longitudinal	EDGE digital health system	AU-ROC	LR	[21]
Exacerbations of COPD patients	Case-control	Equi-ripple bandpass (BP)	Sensitivity, specificity, accuracy, PPV, NPV	PCA coupled SVM	[22]
Diabetes classification	Case study	Age and clinical data	Sensitivity, specificity, accuracy, AU-ROC	LR, ANN, NB, KNN, and RF	[23]
Glomerulus filtration rate estimation	Retrospective cohort study (RCT)	Age, sex, and serum creatinine 99mTc-DTPA imaging	Accuracy	ANN, SVM	[24]
Asthma exacerbations events	Case-control	Telemonitoring data	Sensitivity, specificity, accuracy	NB, adaptive Bayesian network, and SVM	[25]
Stage of lung cancer	Prospective cohort study	Cyrano’s 320 sensor device, age	Accuracy, sensitivity, and specificity	SVM	[26]
Pulmonary function tests	RCT	Blood analysis, lung images	Accuracy	DT	[27]
Dementia prediction	Case-control	MRI	Accuracy, precision, and specificity	SVM	[28]
Identification of ischemic stroke lesions	Cross-sectional	MRI	Accuracy	NB	[29]
Course of depression	Case study	A shortened version of the IDS (QIDS)	Accuracy	LR	[30]
Late-life dementia assessment	Prospective cohort	MRI/CT, Blood Tests	ROC, AUC and, MCA	SVM	[31]
Degenerative movement disorders	Cross-sectional	Pathological	Not defined	Hierarchical clustering analyses	[32]
Checking CT imaging effectiveness	Case study	CT images, Age, and sex	Accuracy, AU-ROC	NN	[33]
Discriminatory peptide identification of heart failures	Experimental	Age, sex, and renal function	Sensitivity, specificity	SVM	[34]
Classification of chronic periodontitis patients	Case-control	Age and PH subjects	Accuracy, Sensitivity, Specificity	SVM	[35]
Classification of fibromyalgia	Case Study	ICD-9 codes	Mean	K-means clustering	[36]
Chronic diseases assessment	Prospective Cohort	Community question answers	Accuracy	NB, SVM, and RNN	[37]

**Table 2:** Pathology types with used models and their strengths and weakness.

Pathology Type	Name	Models	Accuracy (%)	Strengths	Limitations	Future Developments
Liver	Hepatic fibrosis stage[16], and chronic hepatitis-B [19]	NB, RF, KNN, SVM, and NN	78.1–82.7	Liver related diseases produce large patient information, metabolomics analyses, and EHR. Deep learning algorithms help in the prediction of liver therapeutic discovery.	There is currently no complete AI system that can able to detect a couple of abnormalities overall through the human body [38].	Further studies are needed to develop an advanced deep learning algorithm to remedy greater complicated medical imaging troubles, along with ultrasound or Positron-emission tomography (PET) [18].
Pulmonary	COPD exacerbation, asthma exacerbation[25], lung cancer stages [26]	Bayesian Network, LR, SVM, NB, and PCA	62.3–76.1	Studies proposed a data-driven methodology that can help to produce COPD predictive models and asthma exacerbations. It would be useful to support both patients and physicians [39].	Even it is less cost of devices like spirometers to check lung functionality but it is not likely to replaced by quantified computed tomography.	It is highly recommended in future studies to incorporate ML models in the predictive analysis [40].
Nervous system	Dementia, Ischemic stroke lesions identification [29], late-life dementia [31], degenerative moment disorders [32]	SVM, LR, NB, RF, Hierarchical clustering analyses, and DSI	69–80	ML studies in Nervous systems can help to improve the diagnosis of Nerve system conditions	AI-based behavioral systems are still in early to understand the discrete behavior of patient chronic conditions	Future AI might be able to represent these features into one cognitive reinforcement-mastering model [41].
Diabetes	Type 2 Diabetes Mellitus [23]	LR, ANN, NB, KNN, and RF	73.2–91.6	These techniques in diabetic studies can be helpful in symptoms recognition, and disease forecasting	Technological advancements in AI need to more effective with large data sets in diabetes prediction [42]	ML applications need to produce facts on big data mining of medical data sets [42,43].
Kidney Diseases	Glomerular filtration rate estimation [24]	ANN, SVM, Regression and ensemble learning	73.1–76.0	Risk prediction can highly effective in kidney diseases	The research gap in the artificial kidney implantation needs to be addressed [44].	Many demanding situations need to be a success before it becomes a fact and a part of medical practice in nephrology.
Disease-related to muscle pains	Fibromyalgia (FM) [36]	KNN	-	In FM class division, K-means clusters can helpful for categorization of pain, clinical procedure usage, and symptom severity	KNN is a self-learner in trained data classification [45].	Future studies are needed to propose feasible algorithms to forecast FM causes.
Heart diseases	peptides for heart failure [34]	NB, and SVM	84–91	Optimized data-driven ML techniques are helped to predict heart diseases that improve total research and preventive care. Also, it will make sure that many people can happily lead a healthy lifestyle	To predict the risk quality of the heart dataset is needed in clinical practice to support high-quality datasets of heart patients.	Scientists’ are needed to propose precise models to predict the risk of heart failures [46]
Infections	Periodontitis [35]	SVM, NN	Not defined	NN and SVM algorithms are useful in the diagnosis and prediction of periodontal diseases	Lack of optimal datasets and model improvements	A computer-aided classification system can be expected to become an efficient and effective procedure for these inflectional diseases [47]

## Acronyms

ML: Machine Learning; AI: Artificial Intelligence; SML: Supervised Machine Learning; DSS: Decision-support systems; CVD: Cardio Vascular Diseases; RTE: Real-Time Electrographs; CHB: Chronic Hepatitis B; NB: Naïve Bayes; RF: Random Forest; ANN: Artificial Neural Networks; RNN: Recurrent neural networks; KNN: K-Nearest Neighborhood; SVM: Support Vector Machine; NN: Neural networks; LR: Logistic Regression; PCA: Principal Component Analysis. DSI: Discrete Smooth Interpolation; FP: False positives; TP: True positives; FN: False negatives; TN: True negatives; AU-ROC: Area under receiver operating characteristics; COPD: chronic obstructive pulmonary disease; PPV: Positive Predictive Value; NPV: Negative Predictive Value. QIDS: Quick Inventory of Depressive Symptomatology; CT: Computerized Tomography; MRI: Magnetic Resonance Imaging; MCA: Multiple Correspondence Analysis; DTPA: Diethylene Thiamine Penta acetate; PET: Positron-emission tomography; EHR: Electronic Health Record.
